# Nursing of Pike-Perch (*Sander lucioperca*) in Recirculating Aquaculture System (RAS) Provides Growth Advantage in Juvenile Growth Phase

**DOI:** 10.3390/ani13030347

**Published:** 2023-01-19

**Authors:** Géza Péter, Jovanka Lukić, René Alvestad, Zoltán Horváth, Zoltán Nagy, András Rónyai, Péter Bársony, Uroš Ljubobratović

**Affiliations:** 1 Research Centre of Aquaculture and Fisheries, Institute of Aquaculture and Environmental Safety, Hungarian University of Agriculture and Life Sciences, Anna-Liget Str. 35, H-5540 Szarvas, Hungary; 2 Laboratory for Molecular Microbiology (LMM), Institute of Molecular Genetics and Genetic Engineering (IMGGE), University of Belgrade, Vojvode Stepe 444a, 11042 Belgrade, Serbia; 3 Nofima AS, Muninbakken 9-13, Breivika, NO-9291 Tromsø, Norway; 4 H&H Carpió Halászati Kft., Kossuth u. 7, H-7814 Ócsárd, Hungary; 5 Faculty of Agricultural and Food Sciences and Enviromental Management, Institute of Animal Science, Biotechnology and Nature Conservation, University of Debrecen, H-4015 Debrecen, Hungary

**Keywords:** pike-perch, larviculture, intensive rearing, growth rate, survival

## Abstract

**Simple Summary:**

High flesh quality, along with a good potential for intensive farming in a Recirculating Aquaculture System (RAS), make the pike-perch (*Sander lucioperca*) a valuable fish species both from the industry and the research point of view. Although high electricity demands have been attributed to it, fish farming in a RAS is recognized as the key strategy for sustainable fish farming, given its minimal usage of natural resources as well as its independence of outdoor weather conditions. Although the older juvenile stages of pike-perch are well adapted to indoor rearing conditions, the larval and early juvenile stages seem to be particularly sensitive to indoor rearing. Switching to the indoor production of pike-perch larvae would support the continuous, weather-independent, production of fish throughout the year. Hence, our study aimed to identify the pros and cons of rearing pike-perch larvae and early juveniles (nursing period) in indoor versus outdoor systems. For this purpose, we compared the effects of pike-perch nursing in both systems on the later growth and survival of the fish. Our results show that, although nursing indoors increases fish mortality, it provides a basis for improved fish growth later in life. This can eventually shorten the period for the production of market-size fish, and, along with the advantages related to the year-round production of larvae, provide higher yields to fish farmers in the long run.

**Abstract:**

This study aimed to estimate the efficacy of two pike-perch juvenile production technologies: exclusive Recirculating Aquaculture System (RAS) culture (the RAS group) and pond larviculture with a transfer to the RAS at the 42nd day post-hatch (DPH). Both direct weaning on dry feed (the Pond-D group) and 10-day gradual weaning using bloodworms (the Pond-B group) after transfer to the RAS were evaluated in pond-nursed fry. Their survival and morphometric indices were monitored after the RAS habituation period (first 10 days), after the 18-day post-habituation period and after an additional 30 days of on-grow. Our results indicate a negative allometric growth of the pond-nursed fish during the nursing period, which was slower (*p* < 0.0001) in comparison to the RAS-nursed fry (16.3 ± 0.4 vs. 17.8 ± 0.7%/day). After transfer, these fish grew faster than the RAS-nursed fry (7.7 ± 0.1, 4.9 ± 0.5 and 6.1 ± 0.6 during habituation, 8.5 ± 0.6, 9.3 ± 0.5 and 6.7 ± 0.1%/day during post-habituation period, in the Pond-B, Pond-D and RAS groups, respectively). However, four weeks afterwards, the RAS-nursed fry were again superior in terms of growth (4.0 ± 0.1, 3.6 ± 0.2 and 4.6 ± 0.2%/day, for the Pond-B, Pond-D and RAS groups, respectively), and this was accompanied by a significantly lower feed conversion ratio in this group. Although the survival of the RAS-nursed fry during the nursing period was lower in comparison to the pond-reared fry (11.3 vs. 67.3%), the RAS seems to provide a long-term growth advantage.

## 1. Introduction

Environmentally sustainable farming is becoming an imperative in aquaculture, especially given the increasing consumer demand for fish and the growing human population [1]. Since climate changes represent a huge threat to fish farming, extensive research is underway to develop climate-resilient fish production technologies, especially in the face of global warming, which is becoming a leading problem of the 21st century [2]. Indoor fish production, using a Recirculating Aquaculture System (RAS), is the major approach that could successfully overcome climate changes while at the same time reducing natural resource (land and water) use [3].

Pike-perch (*Sander lucioperca*) has been identified as a species with high market potential, due to its high nutritive quality and potential for intensive production [4]. However, with the exception of several Western European farms, current pike-perch production is principally performed outdoors, particularly in the larval stages, because of the high mortality and costs associated with RAS larviculture [5]. There are numerous research data describing the two larval rearing technologies for this species—intensive culture exclusively [6,7,8] and extensive pond nursing followed by dry feed habituation in intensive conditions [9,10,11]. Continuous supply of the grow-out material continues to be the main reason for production stagnation in the latter rearing technique [12,13,14]. Therefore, technological development in RAS larviculture is an ongoing issue from the perspectives of nutrition [7,15,16] and technological solutions [4,8].

Despite both juvenile production technologies seeming to be rather thoroughly explained from both biological and technological perspectives, research evaluating the long-term effects of different conditions during larval pike-perch rearing is limited. Namely, after a period of extensive pond nursing, fish are subjected to the RAS adaptation period. This transition period may provide a short-term advantage or disadvantage, which may extend to the later on-grow phase. Therefore, describing each of the phases separately (RAS/pond nursing, RAS adaptation and post-adaptation of pond-nursed juveniles) may provide a more accurate prediction of fish long-term performance and give a broader view of the pros and cons of each technology. Accordingly, the aim of present study was to describe the production characteristics of each step of the above two pike-perch juvenile production technologies - from RAS adaptation and post-adaptation to the on-growing phase. This modular approach will eventually aid the estimation of the impacts of various larviculture conditions on later fish performance.

## 2. Materials and Methods

Due to the complexity of the work, this section contains only a brief outline of the methodology, while the rest of the details are provided in the Supplementary Materials. Fish handling was performed according to the regulations of the Animal Ethical Panel of the Research Center of Fisheries and Aquaculture (HAKI) at the Hungarian University of Agriculture and Life Sciences (MATE), which was established according to Hungarian state law (10/1999.I.27). 

### 2.1. Fish Origin 

**Figure 1:**
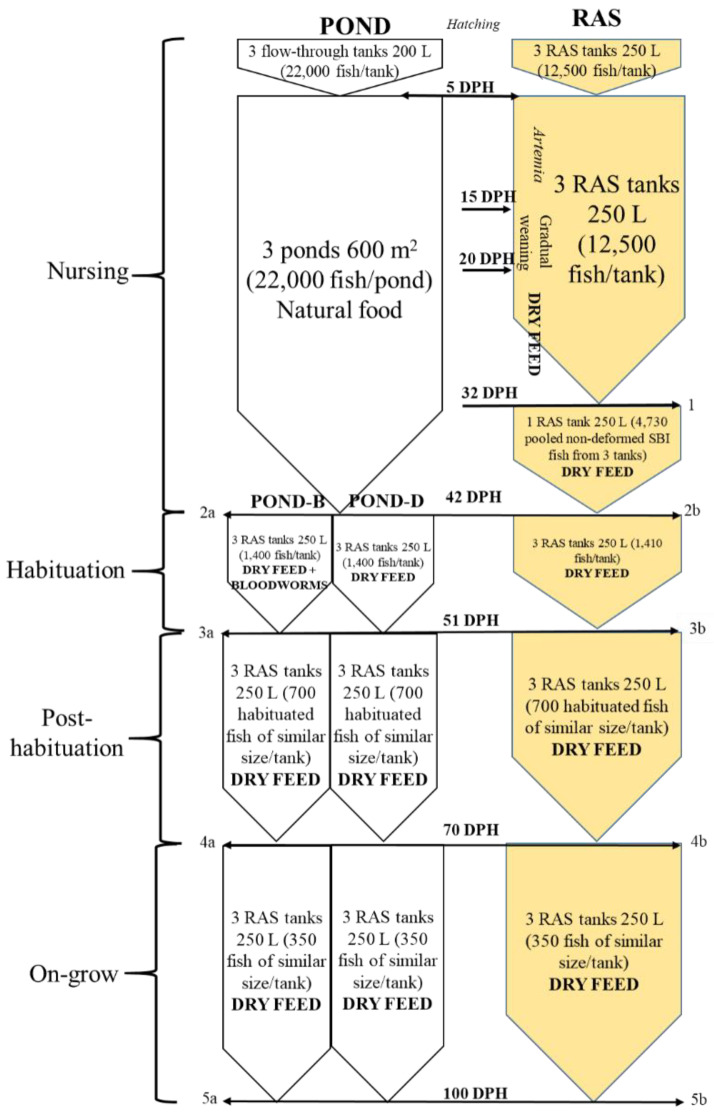
Flow-chart with experimental design, showing four growth/rearing phases assessed in this research: larval nursing, dry fed habituation, post-habituation and the on-growing phase. Fish sampling was performed at five points throughout the experiment: point **1** (when fish were counted and swim bladder inflation (SBI) and deformity rates estimated, along with morphometric indices in all fish), **2** (when fish were counted and morphometric indices assessed in a total of 40 fish from pond (**a**) and 30 fish from RAS (**b**)), **3** (when habituated fish were counted and morphometric indices assessed in 30 fish/tank both from pond- (**a**) and RAS- (**b**) nursed fish), **4** (when fish were counted and morphometric indices estimated in all fish both from pond- (**a**) and RAS- (**b**) nursed groups) and **5** (when fish were counted and morphometric indices estimated in 100 fish/tank both from pond- (**a**) and RAS- (**b**) nursed groups).

The fish used in the present study are products of the artificial reproduction of wild pike-perch breeders from the oxbow of the river Körös. Propagation took place in April 2017 according to the protocol described in Ljubobratović et al., 2019 [17]. Upon hatching, the larvae were pooled and stocked in six tanks with the up-welling flow, three being in the larval nursing RAS and the other three in the flow-through hatchery. In total, 12,500 and 22,000 per replicate volumetrically counted larvae were stocked in the RAS and flow-through hatchery tanks, respectively. On the 5th day post-hatch (DPH), when mouth opening was first noticed, the larvae from the flow-through tanks were stocked in three 600 m^2^ larval nursing ponds (Figure 1). 

### 2.2. Nursing Period 

This rearing stage included both larval (32 DPH [18]) and early post-larval rearing period (up to the 42nd DPH).

#### 2.2.1. Pond

Prior to filling the ponds, 250 kg (4 t ha^−1^) of cow manure was distributed over the base of the pond. On the 42nd DPH, fish were harvested from the ponds and counted. At the day of harvest, measurements of individual total length and bodyweight were assessed for 40 juveniles. 

#### 2.2.2. RAS

The cultivation of larvae in the RAS was carried out according to the protocol described in Ljubobratović et al., 2019 [19]. Larvae were reared in three rearing tanks (250 L). The feeding started at the 5th DPH using freshly hatched *Artemia franciscana* nauplii (AF origin, INVE, Dendermonde, Belgium) until the 7th DPH and *Artemia salina* nauplii (Great Salt Lake (GSL) origin, INVE, Dendermonde, Belgium) from the 8th–15th DPH. At the start of feeding, the larvae were given *Artemia* in the amount of 100 nauplii/larva/day (considering the initial stocking density), and this amount was increased gradually to 300 nauplii/larva/day at the 11th DPH. From the 16th DPH onwards, gradual weaning began, with a daily reduction of the *Artemia* quantity by 20%, using Otohime larval feed (Marubeni Nisshin Feed Co., Japan) (Otohime B1, 250–360 μm from the 16th–18th DPH and Otohime B2, 360–640 μm, from 19th–20th DPH) as a replacement feed. After a total transition to dry feed at the 21st DPH, Otohime B2 was used until the 25th DPH and Otohime C1, 580–840 μm from the 26th–32nd DPH (the end of larviculture). Dry feed was administered using an automatic belt feeder, and the amount ranged from 20 g/tank at the 16th DPH to 70 g/tank at the 32nd DPH (gradual daily increase). At the 32nd DPH, the fish were counted and evaluated for deformities, swim bladder inflation and morphometric indices. All non-deformed, swim bladder inflated (SBI) fish were stocked in a common tank for the following 10 days and reared in the same conditions as the 26th–32nd DPH larvae (Figure 1).

### 2.3. Habituation Period—Transport of Pond-Nursed Juveniles to RAS

At the 42nd DPH, the pond-nursed juveniles were stocked in six randomly chosen tanks (1400 fish per tank), making it 5.6 fish/L as recommended by Policar et al., 2013, and Ljubobratović et al., 2016 [11,20]. Since only a small proportion of the fish from the ponds had to be drawn for further experiments, in order to minimize the sampling errors, the fish from the three ponds were pooled before introduction to the RAS. This way, the margin of error was reduced from 2.5% (95% Confidence Interval (CI)) for sampling 1400 fish from each pond (containing on average 14,300 fish in the case of 65% survival, which was the average survival in our hands) to 1.4% for drawing 3 × 1400 fish from a pooled sample of 3 × 14,300 fish. (https://www.qualtrics.com/experience-management/research/margin-of-error/ (accessed on 23 December 2022). After pooling, the fish were counted, and the individual lengths and weights were estimated for a random sample of 40 juveniles. At the same time, the surviving fish from the RAS (pooled at the 32 DPH, the same as for the pond-nursed fish) were counted (4230 totally) and distributed into three tanks (1410 fish per tank). Individual weights and lengths of the RAS-nursed juveniles were estimated for a random sample of 30 fish (Figure 1).

Three tanks with pond-nursed juveniles were supplied with the dry feed Otohime C2, which was given via an automatic belt feeder (4305 FIAP belt feeder; Aquacultur Fishtechnik, Germany) every 5 min and was the only feeding provided in the tanks making up the group Pond-D. The same feeding methodology was utilized for the groups stocked with RAS-nursed fish (RAS). The feeding rate was 10%. Finally, three tanks of the group Pond-B were submitted to a dry feed habituation protocol including frozen chiromonid according to earlier descriptions [10,11]. The feeding regime during the habituation protocol was as follows:

42 DPH—stocking into the RAS; evening feeding with 3% bloodworms of total biomass;

43 DPH—feeding with 20% bloodworms of total biomass in four meals;

44–45 DPH—feeding with a feed mixture composed of bloodworms and C2 in a 3:1 ratio for a total amount of 20% of the fish biomass (15% bloodworms and 5% C2);

46–47 DPH—feeding with a feed mixture composed of bloodworms and C2 in a 1:1 ratio for total amount of 20% of the fish biomass (10% bloodworms and 10% dry feed);

48–49 DPH—feeding with a feed mixture composed of bloodworms and C2 in 1:2 ratio for total amount of 15% of the fish biomass (5% bloodworms and 10% C2);

50–51 DPH—feeding with dry C2 exclusively in the amount of 12.5% of the fish biomass. 

Thus, in total, three experimental groups were formed and further on monitored in triplications: 

RAS—fish raised in the RAS from hatching onwards;

Pond-D—fish pond-nursed from 5 to 42 DPH and then directly weaned to dry diet;

Pond-B—fish pond-nursed from 5 to 42 DPH and then gradually weaned to dry diets using bloodworms. 

At the 52nd DPH, the fish were harvested from each tank and separated into three fractions [10,21]: (1) starved fish—with obvious signs of starvation, (2) big fish—dramatically larger compared to the majority of the population (assumed cannibals) and (3) habituated fish—without clear signs of starvation and in a size range similar to that of the majority of the population. Only the habituated fish were counted. Mortality was assessed according to the number of dead fish during the period, while cannibalism was calculated based on the number of missing fish. Thirty habituated fish from each of the tanks were measured for their individual wet weight and for their total length.

### 2.4. Post-Habituation Period—After Transition of Pond-Nursed Juveniles to the RAS

Following the habituation period and directly after fish counting, the third period, post-habituation, was initiated at the 52nd DPH (Figure 1). For this purpose, 700 habituated fish from each POND tank were stocked back following counting. Likewise, in the RAS group, 700 fish per tank were stocked back after the selection was conducted in such a manner as to reduce the size variation (simulating size grading). The water flow in all tanks was set at a 150% exchange per hour. During this period, the fish were fed dry feed exclusively. The feeding rate was set at 10%. The fish were fed with Coppens Top (Alltech Coppens Co., Germany) of sizes 0.8–1.2 and 1.2–1.5 mm in the first and second halves of the period, respectively. After 18 days of rearing, on the 70th DPH, all the fish were harvested from the tank. Survival was based on the total number of harvested fish, while cannibalism was calculated based on the number of missing fish. 

### 2.5. Juvenile On-Grow Period

Juvenile on-grow was initiated at the 70th DPH (Figure 1). Straight after post-habituation, 350 fish from each tank were selected. The selection method aimed to minimize the size variation within the tank. Fish were fed Aller Aqua feeds (Aller Aqua, Denmark), Futura EX 1.5 mm pellets and NOVA EX 2 mm pellets, in the first and second halves of the rearing period, respectively. The gradual shift of the feeds was accomplished in three days. After 30 days of rearing, on the 100th DPH, all the fish were harvested from the tank. One hundred fish from each tank were measured for their individual wet weight (±0.05 g) and for their total length (±0.1 mm). 

### 2.6. Data Analysis 

Fish weight, length, mortality and survival were observed directly at the above detailed timepoints, while other indices (cannibalism, specific growth rate (SGR), feed conversion ratio (FCR), coefficient of variation (CV), allometric coefficient (b) and relative condition factor (Kn)) were calculated using the accepted formulas. The specific growth rate (SGR) was calculated using the formula SGR = 100 × (LN(Wf) − LN (Wi))/Δt (Wf = final weight (g), Wi = initial weight (g), Δt = duration of the analyzed period in days) [22]. The allometric coefficient (b) was estimated as the slope of the Length–Weight relationship (LWR): LN (W) = a + b log (L) (W = fish weight in grams, L = fish length in cm, a = the intercept of the regression line, b = slope of the regression line). In order to account for the differences in the growth patterns present in the treatment groups (non-isometric growth), the relative condition factor (Kn) (Kn = Wo/Wc (W0 = observed weight, Wc—calculated weight using LWR)) was used to estimate fish condition [23]. The feed conversion ratio (FCR) was calculated from the post-habituation phase onwards (when all treatment groups were given only dry feed) using the formula FCR = F/ΔB (F = given feed amount in g, ΔB = biomass gain in g) [22]. The in-tank weight coefficient of variation (CV) (CV = SD/Wa (SD = standard deviation, Wa = average weight) [22] was determined at the beginning and end of each monitoring phase in order to estimate the efficacy of the fish grading. The cumulative biomass per larval tank after the on-grow phase was calculated assuming the initial amount of 12,500 larvae and cumulative survival values. During the larval period, comparisons between the two groups (RAS and pond-reared) were made using a *t*-test (IBM SPSS Statistics 21 [24]), except for (1) the comparison of the allometric coefficients (b), which was made using analysis of covariance (ANCOVA) (IBM SPSS Statistics 21), setting LN (weight) as a dependent variable, LN (length) as a fixed factor and treatment (RAS/pond) as a covariate; and (2) the comparison of survivals, which was performed using a chi-square test. SGR were compared using average and SD values (https://www.medcalc.org/calc/comparison_of_means.php (accessed on 16 November 2022)).

During the habituation, post-habituation and on-growing periods, comparisons were made using analysis of variance (ANOVA) and Tukey post-hoc (IBM SPSS Statistics 21), with the average tank values as individual samples (since the treatments were conducted in triplicates with three tanks per treatment group). The comparison of the average CVs between different phases was performed using a paired *t*-test. Since the initial stocking density was shown to affect fish SGR and FCR [25,26], ANCOVA was performed to analyze the effect of stocking density (set as a covariate in the model) in each period. If the effect was significant (*p* < 0.05), the analysis was continued using ANCOVA (Bonferonni post-hoc); otherwise, the analysis was performed using regular ANOVA and Tukey post-hoc. The data throughout the text and in tables are presented as means ± standard deviation (S.D.).

## 3. Results

### 3.1. Larval and Early Post-Larval Rearing 

From the initial 37,500 fish from the RAS group, distributed in three tanks (each with 12,500 fish), only 11,592 fish survived up to the 32nd DPH, from which 4730 had swim bladders and did not show any obvious signs of deformation. The average weight of these fish (only the SBI and non-deformed fish) was 147.56 ± 40.9 mg, and the average length was 25.36 ± 2.4 mm (Table 1), with SGR 17.6 ± 1.1%/day. During the additional 10-day rearing period, the SGR increased to 18.5 ± 4.7%/day, but the survival additionally decreased to 4,230 fish, giving a final 11.28% of surviving RAS-reared juveniles (considering only the SBI and non-deformed fish), in contrast to 67.27 % of surviving juveniles from pond culture. Statistical analysis (*t*-test) supported a significantly higher weight, length, SGR and allometric coefficient for the RAS-reared juveniles in comparison to the pond fish, but a significantly lower survival percentage in comparison to the pond-reared juveniles. The CVs of fish in-tank/pond weight at the 42nd DPH for the RAS and pond groups were 26.9% and 14.8%, respectively.

### 3.2. Habituation Period

After distribution of fish in tanks, CVs at the beginning of the habituation phase were 24.8 ± 1.4, 22.5 ± 5.1 and 27.1 ± 7.2% in the Pond-B, Pond-D and RAS group, respectively. Morphometric estimates and survival counting for Pond-B and -D groups were performed only on habituated fish, as explained above. According to ANCOVA, no significant effect of initial stocking densities on fish SGR was observed (2.5 ± 0.1, 2.4 ± 0.1 and 5.2 ± 0.4 g/L for Pond-B, Pond-D and RAS group, respectively), so the comparisons between the growth rates of the three groups was performed using ANOVA. As provided in Table 2, Pond-B group was presented with the highest growth rate, while the RAS fish had the highest final weights and lengths, as well as the highest survival and the lowest cannibalism percentages in comparison to other treatments. However, cumulative survival of the RAS-nursed group was lower in comparison to pond-nursed groups. CVs at the end of this phase were 36.5 ± 3.9, 36.5 ± 5.4 and 31.8 ± 4.8% for Pond-B, Pond-D and RAS groups, respectively.

### 3.3. Post-Habituation Period 

After grading the fish, CVs at the beginning of this phase were 20.4 ± 1.1, 21.3 ± 0.8 and 21.4 ± 0.8% for Pond-B, Pond-D and RAS group, respectively. Average initial CV at the beginning of the post-habituation period was significantly lower in comparison to final CVs at the end of habituation phase (21 ± 1% vs. 35 ± 5 %, *p* < 0.0001). Similar to the previous phase, initial stocking densities (2.9 ± 0.0, 2.1 ± 0.1 and 5.1 ± 0.1 g/L for Pond-B, Pond-D and RAS group, respectively) did not affect the SGR and FCR of fish during the post-habituation stage. ANOVA revealed significantly lower growth rate, but higher final weights and survival of the RAS-reared juveniles in comparison to both Pond-B and -D fish (Table 3). As for the habituation phase, cumulative survival of the RAS-nursed group was lower in comparison to the other two groups. At the end of the post-habituation period, CVs in the Pond-B, Pond-D and RAS group were 33.1 ± 4.1, 32 ± 6.5 and 27.1 ± 3.2%, respectively.

### 3.4. On-Growing Period

At the beginning of the on-growing phase, the CVs in the Pond-B, Pond-D and RAS group were 18.1 ± 1, 18.8 ± 0.6 and 20.2 ± 1.5%, respectively. The average CV at the beginning of the on-grow phase was significantly lower in comparison to the final CV at the end of the post-habituation phase (19 ± 1 % vs. 31 ± 5%, *p* < 0.0001). During this period, the stocking densities (6.8 ± 0.3, 6.1 ± 0.4 and 7.6 ± 0.4 g/L, in Pond-B, Pond-D and RAS group, respectively) significantly affected the SGR of the fish (*p* = 0.009) and FCR (*p* = 0.007), according to ANCOVA. Therefore, the SGR and FCR were compared controlling for the initial stocking density, while the comparisons for other growth parameters were performed using regular ANOVA. The analysis revealed significantly higher SGR, final weights and final biomass, but lower FCR, for the RAS-reared juveniles in comparison to both the Pond-B and Pond-D groups (Table 4). Similar to the previous two phases, cumulative survival of the RAS-nursed fish was lower than that of the pond-nursed groups. Cumulative biomass (per 12,500 larvae) was also the lowest in the RAS group. The percentage of gill cover deformities in the RAS group at the end of the on-grow phase was 16.7 ± 8.8%, the scoliosis percentage was 2.2 ± 1.9% and the kyphosis percentage was 1.1 ± 1.9%, while no deformities were observed in the other two groups. Gill cover deformity percentage was significantly higher in the RAS group in comparison to both the Pond-B (*p* = 0.017) and Pond-D (*p* = 0.017) groups, while no significant differences were observed for scoliosis and kyphosis between the treatment groups. At the end of the on-growing phase, CVs were 22.6 ± 3.8, 24.5 ± 2.9 and 23.8 ± 1.7% in the Pond-B, Pond-D and RAS groups, respectively. 

## 4. Discussion

Given the rapid accumulation of knowledge from the research on intensive (RAS) pike-perch larviculture [4,27,28,29], integrated comparisons between the pond and the RAS should be performed, in order to incorporate the achievements of science into industry in a timely manner and thus accelerate the development of sustainable systems in aquaculture. This research, accordingly, aimed to evaluate the current advantages and/or disadvantages of pike-perch RAS larvi/early juvenile culture over pond culture. Our results showed that, although the survival of the RAS-produced juveniles at the 42nd DPH was lower, these fish grew faster than the pond-reared fish. According to the results provided in Section 3.1, the fast growth of the RAS fish probably occurred in the post-larval period, after 32 DPH. A regression analysis of the length–weight relationship (LWR) at the 42nd DPH showed that the growth pattern of the fish reared in the RAS was isometric, which is considered to be the optimal growth pattern for juvenile fish, established after the notochord flexion stage (which in pike-perch occurs at around the 17th DPH) [29,30]. On the other hand, the pond-reared fish presented a negative allometric growth at the 42nd DPH. This may have indicated differences in food availability between the two larval rearing systems [31]. However, additional factors with long-term effects may have affected the growth of the fish nursed in the RAS and pond, as discussed below. Interestingly, moving of the fish from the pond to the RAS at the 42nd DPH restored isometric growth, and these fish experienced a fast growth rate during the RAS habituation and post-habituation periods (the first four weeks after the transfer to the RAS), presumably as the result of compensatory growth after the food restriction and resupply [32]. This was particularly pronounced in the pond-reared fish subjected to slow weaning (adaptation to dry feed using bloodworms). However, after the end of the RAS habituation and the immediate post-habituation period, the RAS-reared fish started again to gain a growth advantage, indicating the long-term benefits of RAS larvi/early juvenile culture on fish growth. These beneficial effects of RAS larviculture in respect to fish growth could be ascribed to three potential mechanisms:

1. Physiological adaptations to the RAS environment, particularly different feeding regimes. This reflects the phenotypic plasticity of larval fish, allowing the fish’s physiology to be shaped by environmental stimuli early in development. Engrola et al., 2009, reported that the early introduction of inert feed to Senegalese sole (*Solea senegalensis*) larvae was linked to better growth at the post-larval stage [33]. This may potentially reflect changes in the digestive system due to different diets in early development. In line with this, differences in digestive enzyme gene expression were observed in pike-perch larvae given different diets [34]. In addition, our group has demonstrated an improvement of the condition of pike-perch juveniles subjected to very early weaning (at the start of exogenous feeding) in comparison to fish weaned at the late larval phase [7]. 

2. Microbial maturation—according to this hypothesis, RAS-rearing water is considered to have a stable nutrient-microbiota balance, which suppresses the growth of fast growing (potentially pathogenic) and supports the growth of slow-growing (potentially beneficial) bacteria [35]. In contrast to the pond, which is overwhelmed with a constant nutrient input which disturbs the balance between fast- and slow-growing microorganisms, in favor of the former [36], the RAS is a microbially mature system, due to the presence of a biofilter outside of the fish rearing units, which is allowed to mature (reach the stage of slow-growing bacteria dominance) at the same nutrient concentration that is present in the fish rearing tanks [35]. Though the disinfection applied in our experiment (tank cleaning twice a day, see Supplementary Materials) presumably lowered the efficacy of microbial maturation in the RAS, long water retention time apparently supported a higher degree of microbial maturity in comparison to the pond [37]. Microbial maturation can later improve the larval performance through a more efficient counteracting of the pathogenic bacteria encountered during subsequent growth stages. Indeed, Deng et al., 2022, demonstrated that *Cetobacterium* was credited with numerous beneficial effects in fishing RAS-reared Nile tilapia (*Oreochromis niloticus*) fry in comparison to fry reared in a classical flow-through system (FTS) [38]. Beneficial effects of *Cetobacterium* have been reported in carp and zebrafish as well [39,40]. Similarly, Vestrum et al., 2018, observed a lower growth of the potentially pathogenic *Arcobacter* in RAS-reared cod (*Gadus morhua*) larvae in comparison to FTS-reared fish [35].

3. Genetic selection. One of the main stressors encountered by fish larvae reared in the RAS, which is rarely present in ponds, is the failure to inflate the swim bladder [41]. Swim bladder non-inflation remains the main culprit of high mortality during intensive larval rearing [42]. The reasons for the failure of swim bladder inflation have not been fully elucidated, but both environmental and intrinsic factors, such as body size and air gulping capability, were shown to influence swim bladder inflation success [43]. This implies that those fish genetically “equipped” to grow faster will be superior in terms of swim bladder inflation in comparison to the others. The role of genetics in swim bladder inflation has already been proposed [44]. It is thus possible that the RAS could act as a strong selective pressure enabling the survival of the fittest individuals predisposed to better growth throughout their growth cycle.

The results of the present study are in contrast with the results obtained in a study performed by Policar et al., 2016 [45], which demonstrated that pond-reared pike-perch larvae were superior in terms of both survival and growth in comparison to RAS-reared fish. Aside from the possible differences which might arise due to the use of different technologies (e.g., feed quality, weaning protocols) in two rearing facilities, we assume that the elimination of swim bladder non-inflated (SBNI) fish and fish with different deformities, which was performed in our experiment at the 32nd DPH, might have affected the growth of conspecifics in the same tank. The differences in the growth rates of RAS-reared fish before and after the elimination of SBNI fish at the 32nd DPH in our experiment were subtle (showing a statistical trend *p* = 0.079), but this step might have potentially offered more benefits in the later growth phases. For instance, Schwebel et al., 2018, demonstrated that California yellowtail (*Seriola dorsalis*) without an inflated swim bladder (SBNI fish) have high energy and oxygen demands [46]. This means the oxygen availability may be different in the presence and absence of SBNI fish, especially as the fish reach larger sizes [46]. In addition, research performed by Barcellos et al., 2011, with jundiá (*Rhamdia quelen*) and Nile tilapia demonstrated an increase in the cortisol levels in fish reared in the same tank as stressed fish [47]. This has been attributed to an ancient communication mechanism among fish, preparing “healthy” conspecifics for an upcoming stressor [47]. Both oxygen availability and cortisol levels could potentially affect the growth of healthy fish and prevent them from reaching their full growth potential during the on-growing period. 

Although cost-benefit calculation is beyond the scope of this study, according to the number of surviving fish (~10% in RAS vs. ~35% in pond groups), the income for selling 10 g of fingerlings would obviously be higher for pond rearing [48]. There are no available data on the comparisons of the costs of pike-perch production between the RAS and the pond. However, a model provided by Kamstra, 2003 [48], was used by Schram et al., 2006, to compare the production costs of closely related perch in the two systems at the same farm [49]. If this model is considered, when correcting for the differences in mortalities obtained in our study for pike-perch (50% in the RAS and 36% in the pond), it can be roughly estimated (by simple multiplication of pike-perch/perch survival ratio by the costs obtained by Schram et al.) that, for the production of 100,000 juveniles (90 DPH) in the RAS, the cost would be 68,000 €, while, for pond production, the cost would be 35,000 €. We note that this is just a simple correction, so more extensive calculations, using the model of Kamstra, 2003 [48], should be performed in order to obtain exact costs. However, rough estimates using the final weights and estimated marginal growth rates from Table 4 indicate that RAS-reared fish could potentially reach the market size (1000 g) approximately 15% faster in comparison to fish from pond larviculture (this is only rough estimation since the growth rates are expected to decrease as the fish grow).This means that, if the market size of approximately 1 kg for RAS-reared pike-perch can be reached in 18 months [50], then, theoretically, the RAS-nursed larvae could attain this size 2.7 months faster (in 15.3 months) in comparison to pond-nursed fish. This means faster access to money which can be used for further investments (570,000 € if the market price per kg is 6 € and the survival rate is 98 %, as obtained in our research for the on-growing stage [48]) and more juveniles produced per year.

Though the costs associated with RAS are significantly higher in comparison to pond rearing, novel eco-friendly techniques for disease control, such as probiotics, can be cost-effectively applied in the RAS. Probiotics applied very early during larval development could offer additional advantages related to nutrient utilization efficacy, which could boost fish growth beyond the levels offered by the RAS itself [7,27,51]. Survival and performance of RAS-reared fish can additionally be improved by the usage of different live foods (e.g., rotifers) as well as the enrichment of live food with polyunsaturated acids or vitamins [16,28,52]. In addition, due to high neural plasticity of fish, RAS offers a multitude of opportunities for controlled fish manipulations which could enhance the fish’s cognitive performance (and potential productivity) later in life [53,54].

Aside from showing the advantages of RAS larviculture in terms of fish growth, our study is among the first to demonstrate a successful RAS transition of pond-nursed fry to dry feed, without bloodworm supplementation. Advancement in this respect is rather limited and is focused mainly in a few research centers in Central and Eastern Europe [9,10,11]. Although, during the habituation and post-habituation periods, the survival and growth of these fish were lower in comparison to the fish weaned using bloodworms, the growth during the on-growing period was not significantly different in comparison to the latter group, suggesting no long-term effects of direct dry feed adaptation. Given the high labor costs associated with bloodworm supplementation [10,11], our research offers an incentive for fish farmers to apply this approach, which, unlike exclusive cultivation in the RAS, is currently accessible and the easier to implement.

## 5. Conclusions

To summarize, this study showed that the RAS can be considered a satisfactory rearing system for larval pike-perch. Although RAS nursing brings with it disadvantages in terms of survival in comparison to pond nursing, it can potentially bring benefits for fish growth in the later rearing phases. Elimination of deformed fish may be a critical point in successful growth of RAS-reared fish. Combined with the benefits that could be provided by modern manipulation tools applied during indoor larviculture (e.g., probiotics, training), it can be assumed that the RAS-produced juveniles have a potential to bring a higher income to fish farmers in comparison to pond rearing. However, additional work is needed before our research results can be incorporated into the practice. First, investigation of possible reasons behind the very high mortality of RAS-nursed fish in the larval phase and identification of measures which could effectively reduce it should be undertaken. In addition, a cost-benefit calculation is needed to give the exact juvenile cost in both systems and the amount of yearly juvenile production which would incur a significant economic advantage for the RAS nursing system, when all variables provided in this research are included.

## Figures and Tables

**Table 1:** Survival and morphometry of pond- and RAS-produced juveniles of pike-perch *Sander lucioperca* at the 42 DPH (mean ± S.D., for morphometric indices n = 30 and 40 fish in the case of RAS and Pond group, respectively). Initial weights of all fish (0 DPH) were approximately 0.5 mg. Results of *t*-test are provided as superscripts.

Parameters	Pond	RAS
Final weight (mg)	469.6 ± 69.4 ^A^	929.18 ± 250.4 ^B^
Final length (mm)	39.4 ± 2.2 ^A^	46.1 ± 3.8 ^B^
Specific growth rate (%/day)	16.3 ± 0.4 ^A^	17.8 ± 0.7 ^B^
Allometric coefficient (b)	2.11 ^A^	3.01 ^B^
Relative condition factor (Kn)	1.00 ± 0.09 ^A^	1.01 ± 0.17 ^A^
Survival (%)	67.3 ^A^	11.3 *^B^

* Only non-deformed fish were included.

**Table 2:** T Survival and morphometry of pond and RAS produced pike-perch *Sander lucioperca* juveniles at the end of the habituation period, on the 52 DPH (mean ± S. D., n = 3 tanks); results of post-hoc tests are provided as superscripts.

Parameters	Pond-B	Pond-D	RAS
Initial weight (g)	0.44 ± 0.02 ^A^	0.43 ± 0.01 ^A^	0.93 ± 0.08 ^B^
Final weight (g)	1.0 ± 0.0 ^A^	0.7 ± 0.0 ^B^	1.8 ± 0.8 ^C^
Final length (mm)	44.5 ± 0.4 ^A^	40.5 ± 0.6 ^B^	53.5 ± 1.2 ^C^
Specific growth rate (SGR) (%/day)	7.7 ± 0.1 ^A^	4.9 ± 0.5 ^B^	6.1 ± 0.6 ^A^
Allometric coefficient (b)	3.04 ± 0.45 **^A^**	3.02 ± 0.36 **^A^**	2.95 ± 0.46 **^A^**
Relative condition factor (Kn)	1.01 ± 0.00 ^A^	1.01 ± 0.00 ^A^	1.01 ± 0.00 ^A^
Cannibalism (%)	24.2 ± 2.4 ^A^	19.1 ± 3.1 ^A^	5.5 ± 0.6 ^B^
Mortality (%)	5.4 ± 1.6	5.4 ± 1.2	4.7 ± 0.5
Survival (%)	70.4 ± 2.9 ^A^	75.5 ± 2.2 ^A^	89.8 ± 0.4 ^B^
Cumulative survival (%)	47.3 ± 1.9 ^A^	50.8 ± 1.5 ^A^	10.1 ± 0.1 ^B^

Pond-B—fish gradually weaned to dry diet using bloodworms; Pond-D—fish weaned directly to dry diet straight upon the transfer from pond to RAS.

**Table 3:** Survival and morphometry of pond and RAS produced pike-perch *Sander lucioperca* juveniles at the end of post-habituation period, 70 DPH (means ± S. D., n = 3 tanks); results of post-hoc tests are provided as superscripts.

Parameters	Pond-B	Pond-D	RAS
Initial weight (g)	1.90 ± 0.03 ^A^	0.74 ± 0.05 ^B^	1.80 ± 0.04 ^C^
Final weight (g)	4.7 ± 0.6 ^A^	4.0 ± 0.1 ^A^	6.0 ± 0.0 ^B^
Final length (mm)	78.5 ± 2.0 ^A^	72.4 ± 1.1 ^B^	81.3 ± 1.0 ^A^
Specific growth rate (SGR) (%/day)	8.5 ± 0.6 ^A^	9.3 ± 0.5 ^A^	6.7 ± 0.1 ^B^
Feed conversion ratio (FCR)	0.72 ± 0.11 ^A^	0.70 ± 0.06 ^A^	0.79 ± 0.02 ^A^
Allometric coefficient (b)	2.77 ± 0.82 ^A^	2.93 ± 0.23 ^A^	3.09 ± 0.27 ^A^
Relative condition factor (Kn)	1.01 ± 0.00 ^A^	1.00 ± 0.00 ^A^	1.01 ± 0.00 ^A^
Cannibalism (%)	19.6 ± 2.3 ^A^	27.2 ± 2.6 ^B^	0.3 ± 0.3 ^C^
Mortality (%)	3.7 ± 1.1 ^A^	5.7 ± 1.2 ^A^	1 ± 0.2 ^B^
Survival (%)	76.7 ± 1.6 ^A^	67.0 ± 3.8 ^B^	98.7 ± 0.2 ^C^
Cumulative survival (%)	36.3 ± 1.5 ^A^	34.0 ± 1.6 ^A^	10 ± 0.1 ^B^

Pond-B—fish gradually weaned to dry diet using bloodworms; Pond-D—fish weaned directly to dry diet straight upon the transfer from pond to RAS.

**Table 4:** Survival and morphometry of pond and RAS produced pike-perch *Sander lucioperca* juveniles at the end of the on-growing period, 101 DPH (means ± S.D., n = 3 tanks). * ANCOVA estimated marginal means evaluated at the stocking density of 6.8 g/L; results of post-hoc tests are provided as superscripts.

Parameters	Pond-B	Pond-D	RAS
Initial weight (g)	4.8 ± 0.1 ^AB^	4.3 ± 0.3 ^B^	5.4 ± 0.3 ^A^
Final weight (g)	16.3 ± 0.7 ^A^	14.5 ± 0.3 ^B^	18.7 ± 0.4 ^C^
Final length (mm)	121.6 ± 1.6 ^A^	116.5 ± 1.3 ^B^	121.9 ± 1.5 ^A^
Specific growth rate (SGR) (%/day)	* 4.0 ± 0.1 ^A^	* 3.6 ± 0.2 ^A^	* 4.6 ± 0.2 ^B^
Feed conversion ratio (FCR)	* 0.72 ± 0.04 ^A^	* 0.86 ± 0.07 ^A^	* 0.54 ± 0.07 ^B^
Allometric coefficient (b)	3.17 ± 0.20 ^A^	3.13 ± 0.04 ^A^	2.87 ± 0.14 ^A^
Relative condition factor (Kn)	1.00 ± 0.00 ^A^	1.00 ± 0.00 ^A^	1.01 ± 0.00 ^A^
Cannibalism (%)	1.4 ± 0.3 ^A^	0.8 ± 0.6 ^A^	1.4 ± 1.8 ^A^
Mortality (%)	0.5 ± 0.3 ^A^	0.3 ± 0.3 ^A^	0.7 ± 0.7 ^A^
Survival (%)	98.1 ± 0.6 ^A^	99.0 ± 0.8 ^A^	97.9 ± 1.7 ^A^
Cumulative survival (%)	35.6 ± 1.6 ^A^	33.7 ± 1.3 ^A^	9.8 ± 0.2 ^B^
Cumulative biomass (kg/12,500 larvae)	72.7 ± 5.0 ^A^	61.2 ± 2.8 ^B^	23.0 ± 0.7 ^C^

Pond-B—fish gradually weaned to dry diet using bloodworms; Pond-D—fish weaned directly to dry diet straight upon the transfer from pond to RAS.

## Data Availability

Raw data from this study are available at ResearchGate next to the abstract of this publication.

## Supplementary Materials

The following supporting information can be downloaded at: https://www.mdpi.com/article/10.3390/ani13030347/s1. Refs. [55,56] are cited in Supplementary Materials.
