# Supplementary material for: Nursing of Pike-Perch (Sander lucioperca) in Recirculating Aquaculture System (RAS) Provides Growth Advantage in Juvenile Growth Phase
Source: Animals (Basel). 2023 Jan 19;13(3):347. doi: 10.3390/ani13030347 (PMC9913090; doi:10.3390/ani13030347)
Supplement: Supplementary file 1 [file animals-13-00347-s001.zip › animals-2074429-supplementary.pdf]

Methodology

2.1. Fish origin

Before each manipulation (weight and length measurements) fish were anaesthetised in 2-phenoxyethanol (300 μL L-1). Eggs of three females were fertilized with the milt of four males. Embryo survival was 75.5 %, 86.9 %, and 89.4 % per female.

2.2 Larval rearing

2.2.1 Pond

Water in ponds was filled through 500 μm mesh to the half of the pond's total depth three weeks prior to stocking of the larvae. Following the stocking of the larvae, water level was increased for 20 cm per week and each week 1 t ha-1 cow manure was added. Finally, the pond was totally filled with water four weeks after stocking. The fish size and homogeneity were monitored once per week from the second week of nursing.

2.2.2 RAS

Rearing tanks has white conical bottoms and black cylindrical walls, equipped with flat fan nozzle sprayer to support swim bladder inflation, as reported in Fazekas et al., 2021 [1]. Water was disinfected using both UV and ozone and treated using bead filter, moving bed bioreactor and trickling tower. Up to the 15th DPH (before inflation of the swim bladder) 5-15 lux light intensity on tank surface and photo period of 14:10 light-dark was used; afterwards, light intensity was increased to 20-40 lux with 24 h light regimen. Feces and uneaten feed were removed from tanks twice a day via cleaning cup set at the bottom of each tank. At the beginning of the trial, water flow in tanks was 30%/h and was increased gradually to 150%/h in the first 25 days of the rearing.

Otohime B composition was as follows: 56.3 % crude protein, 15.9 % crude fat, 2.6 % crude fibre, 13.5 % crude ash, 2.5 % Ca, 2.3 % P) while Otohime C1 composition was: 58.3 % crude protein, 12.9 % crude fat, 1.6 % crude fibre, 15 % crude ash, 2.7 % Ca, 2.5 % P.

Rearing water quality at the outflow of tanks and pond was assessed from 1st to 42ndDPH. Mean water oxygen saturation (daily measurements) was 134.4 ± 13.5 % and 100.9 ± 5.6 % in ponds and tanks, respectively, while the mean temperature (daily measurements) was 17.6 ± 1.1°C in tanks and 17.8 ± 0.3°C in ponds. Mean ammonium-nitrogen, nitrite-nitrogen and nitrate-nitrogen content (measurements twice a week) were 0.19 ± 0.09 mg L-1 (max. 0.45), 0.05 ± 0.02 mg L-1 (max. 0.08), 1.15 ± 1.07 mg L-1 (max. 3.06) in ponds, while in the rearing tanks these values were 0.15 ± 0.07 mg L-1 (max 0.28), 0.04 ± 0.02 mg L-1 (max 0.07) and 13.9 ± 3.6 mg L-1 (max 18.7), respectively.

2.2 Habituation period - transport of pond-nursed juveniles to RAS

Water flow in all nine rearing tanks was set on 150% exchange per hour (~6 L/min). Cleaning of tanks was performed twice per day and dead fish were counted at each cleaning occasion.

Water oxygen saturation and temperature were monitored daily on the outflow of each rearing tank. Mean water oxygen saturation was Pond-D 114± 9.5 %, Pond-B 107.3 ± 8.6 %, RAS 98.0±8.5 %, while the mean temperature

was 22.9 ± 0.4°C for all groups. Ammonium–nitrogen, nitrite–nitrogen and nitrate–nitrogen content in the outflow water were 0.18 ± 0.05 (max 0.22), 0.10 ± 0.03 (max 0.12) and 25.45 ± 9.55 (max 32.2) mg L-1, respectively.

2.3 Post-habituation period - after transition of pond-nursed juveniles to RAS

Feeding was applied mechanically via mechanical belt feeder (4305 FIAP belt feeder; Aquacultur Fishtechnik. Germany) constantly, while the light regime was set on 24:0 LD with 10 lux light intensity on the surfaceon surface of the tanks [2, 3]. Cleaning of the tanks was performed once per day each morning when the number of dead fish was assessed. Water temperature and water oxygen satiation were checked daily in the sample taken at the outflow of each tank.

Water oxygen saturation and water temperature were monitored daily on the sample taken from outflow of each rearing tank. Mean water oxygen saturation was 130 ± 8.1 %, 121.3 ± 7.7 %, and 113.2 ± 8.7 %, in Pond-D, Pond-B and RAS, respectively, while the mean temperature in all tanks was 23.1 ± 0.1 °C. Ammonium–nitrogen. nitrite–nitrogen and nitrate–nitrogen content in the outflow water were 0.31 ± 0.18 (max 0.60), 0.11 ± 0.04 (max 0.17) and 53.64 ± 15.81 (max 73.1) mg L-1, respectively.

2.4 Juvenile on-grow period

Water flow in all tanks was maintained at 150% exchange per hour, while the feeding rate was set at 5.5%. Feeding was applied mechanically via mechanical belt feeder (4305 FIAP belt feeder; Aquacultur Fishtechnik, Germany) constantly, while the light regime was set on 24:0 LD with 5 lux light intensity on the surface of the tanks. Cleaning of the tanks was performed once per day each morning when the number of dead fish was assessed.

Water oxygen saturation and temperature were monitored daily on the outflow of each rearing tank, while the other water quality parameters were assessed twice per week at the common outflow tank. Thus, mean water oxygen saturation was 115.1 ± 13.4 %, 114.2 ± 12.0 %, 113±13.6 %, for the Pond-D, Pond-B, and RAS, respectively, while the mean temperature for all tanks was 23.2 ± 0.1°C. Ammonium–nitrogen, nitrite–nitrogen and nitrate–nitrogen content in the outflow water were 0.21± 0.03 (max 0.25), 0.14 ± 0.03 (max 0.17) and 90.92 ± 14.81 (max 107) mg L-1, respectively.

References

1. Fazekas, G.; Vass, V.; Demény, F.; Tóth, F.; Ljubobratović, U. The effect of different surface-cleaning devices on the success of swim bladder inflation in zander larvae. *N. Am. J. Aquac.* **2021**, 83, 78-82. https://doi.org/10.1002/naaq.10172
2. Kozłowski, M.; Zakęś, Z.; Szczepkowski, M.; Wunderlich, K.; Piotrowska, I.; Szczepkowska, B. Impact of light intensity on the results of rearing juvenile pikeperch, *Sander lucioperca* (L.), in recirculating aquaculture systems. *Fisheries & Aquatic Life* **2010**, 18: 77-84. https://doi.org/10.2478/v10086-010-0009-9
3. Luchiari, A.C.; De Morais Freire, F. A.; Koskela, J.; Pirhonen, J. Light intensity preference of juvenile pikeperch *Sander lucioperca* (L.). *Aquac. Res.* **2006**, 37: 1572-1577. https://doi.org/10.1111/j.1365-2109.2006.01599.x
